# Supplementary figures and images for: MouseNet: A biologically constrained convolutional neural network model for the mouse visual cortex
Source: PLoS Comput Biol. 2022 Sep 6;18(9):e1010427. doi: 10.1371/journal.pcbi.1010427 (PMC9481165; doi:10.1371/journal.pcbi.1010427)

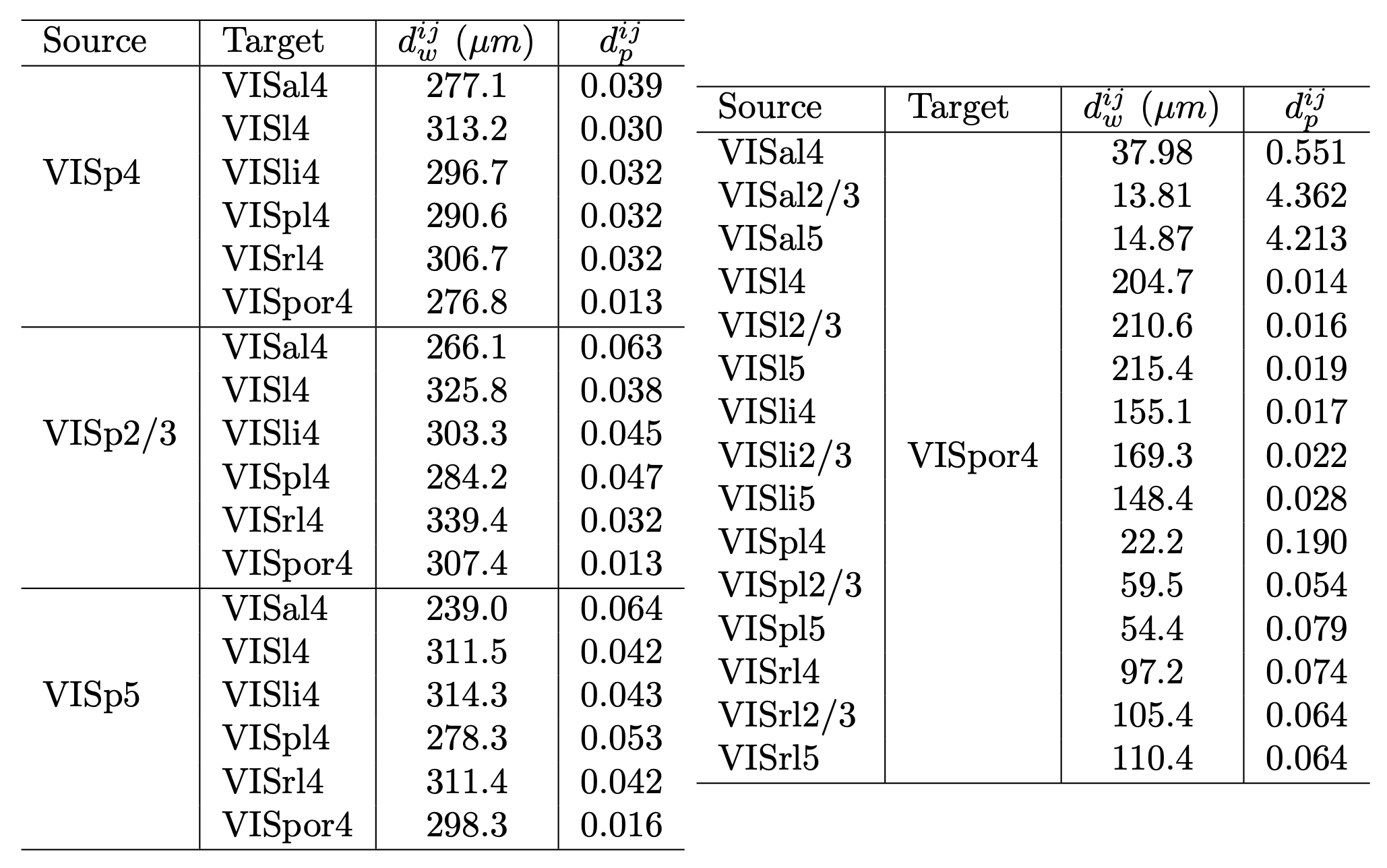

Supplement: S1 Table — (TIF) [file pcbi.1010427.s001.tif]

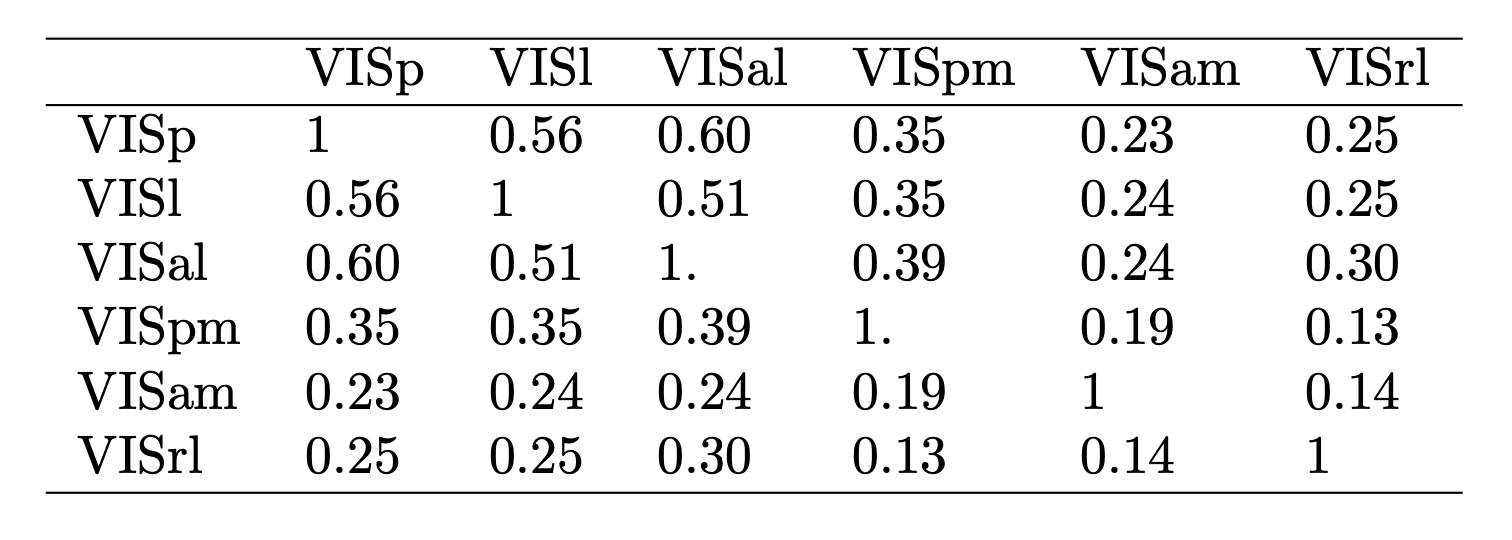

Supplement: S2 Table — Note that even with the neural sub-sampling issue [29], the similarity values between VISp, VISl, and VISal are much higher than they are with the CNN models. (TIF) [file pcbi.1010427.s002.tif]

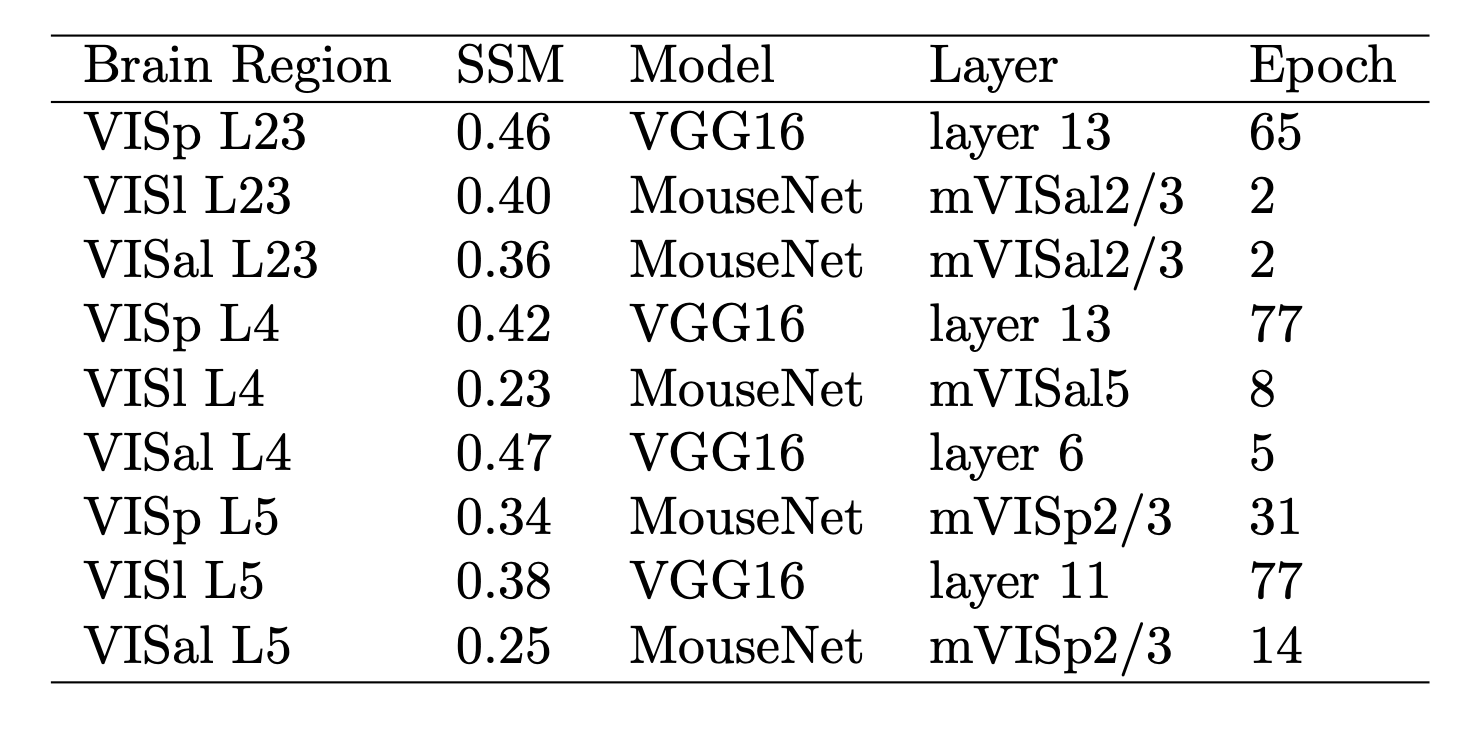

Supplement: S3 Table — (TIF) [file pcbi.1010427.s003.tif]

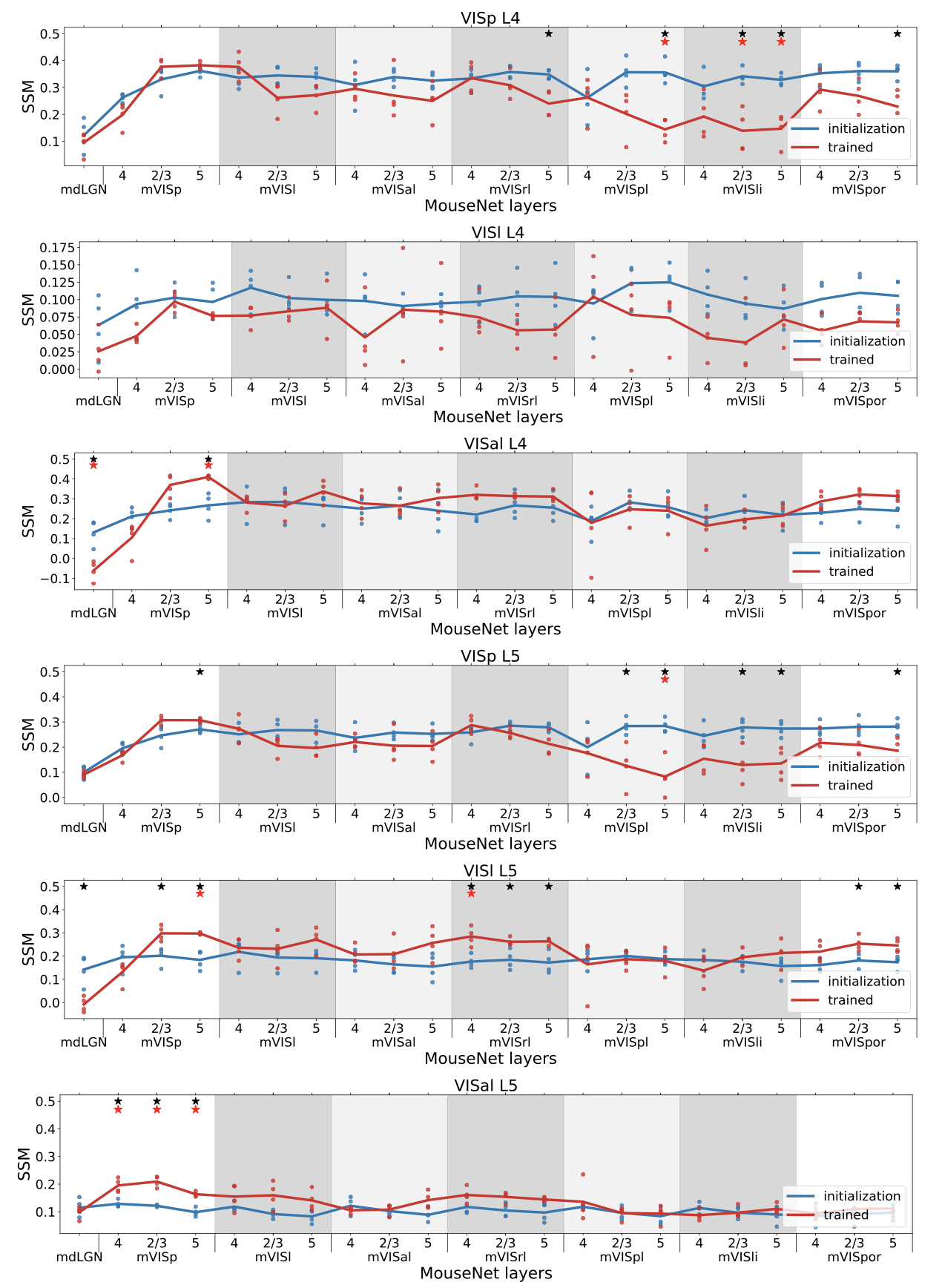

Supplement: S1 Fig — Each line corresponds to the mean of 4 different MouseNet instances trained from different initialization weights (dots). The x axis includes all the layers in the model in a serial way. The five parallel secondary visual area pathways in the model are in shaded grey background. Black stars denote the the pvalues of two-sample t-test with Benjamini/Hochberg correction of 22 comparisons within one brain area is less than 0.05; Red stars denote the pvalues of two-sample t-test with Benjamini/Hochberg correction of all 9x22 comparisons across all 9 brain areas is less than 0.05.). (TIF) [file pcbi.1010427.s004.tif]

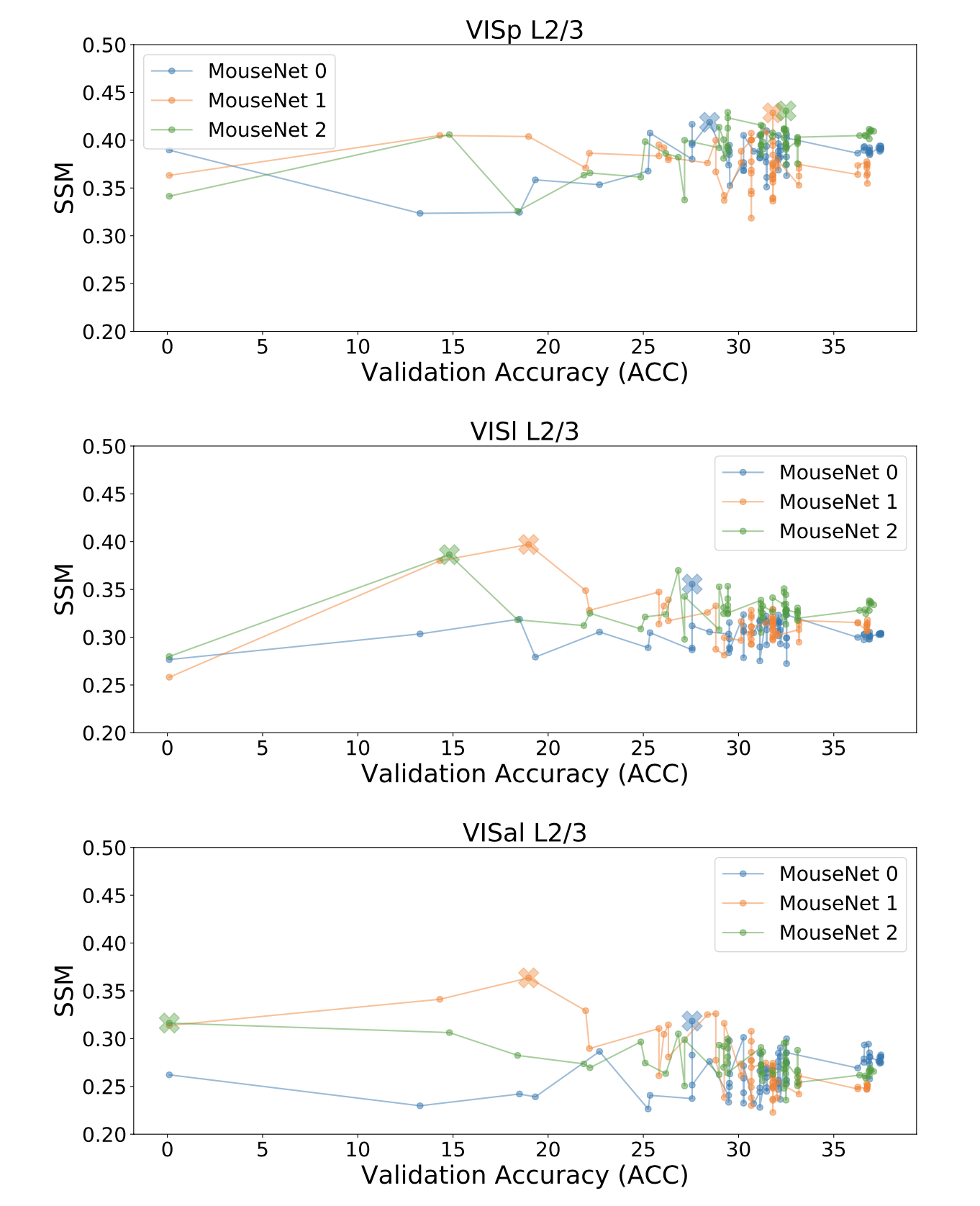

Supplement: S2 Fig — Each row compares models with a different brain area. We show three instances of MouseNet during their training process. Each dot represents the best layer’s SSM of one instance at a certain epoch to the specified brain area, with each instance’s highest achieved SSM during training process marked by a cross. The clear jumps of validation accuracy occurred when we reduced the learning rate. (TIF) [file pcbi.1010427.s005.tif]

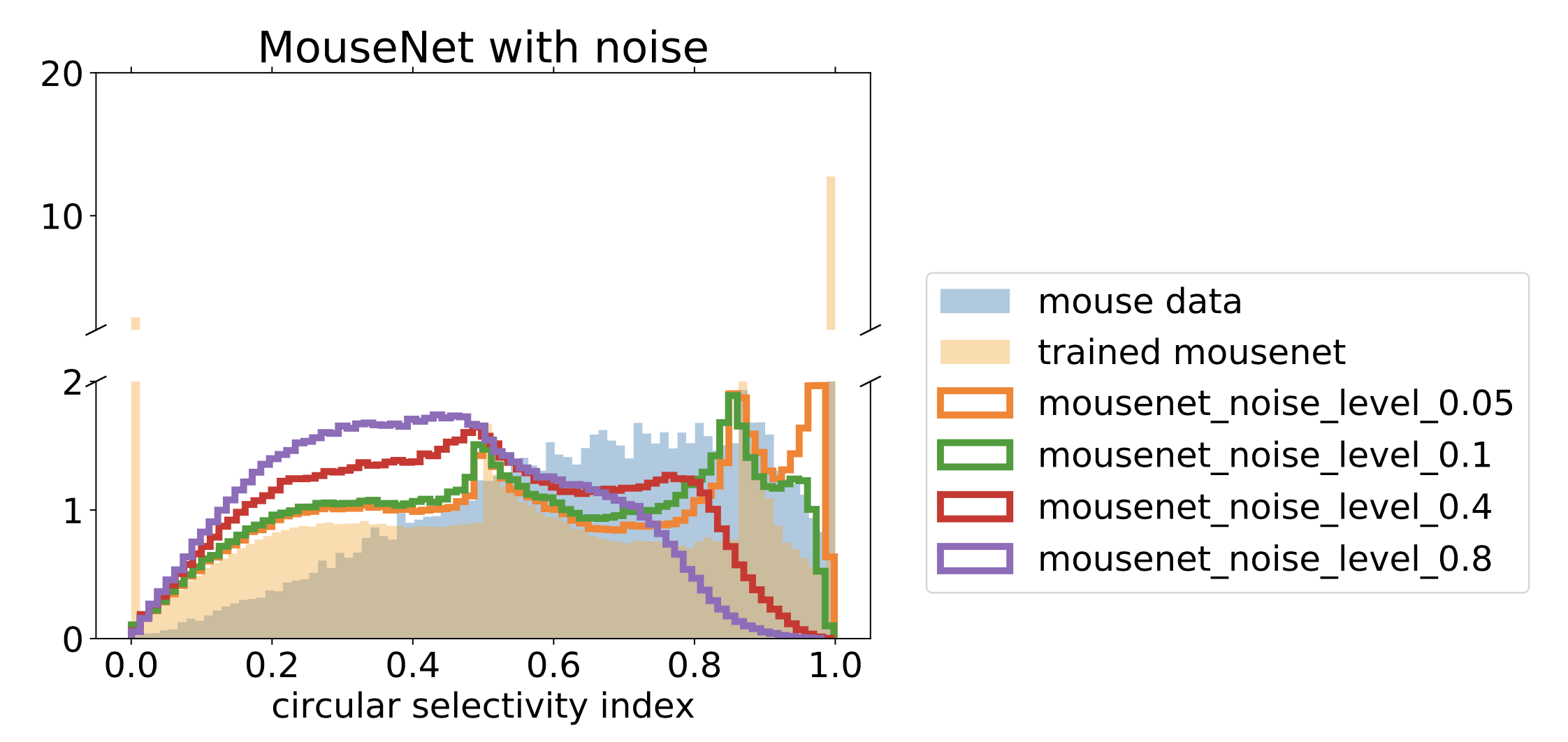

Supplement: S3 Fig — The noise is added to the activations of each layer as a half-normal distribution with a standard deviation of the specified noise level multiplied by the mean activation across all units for that layer. This results shows that circular selectivity index distribution can be smoothed out by adding noise to the deterministic MouseNet model. (TIF) [file pcbi.1010427.s006.tif]

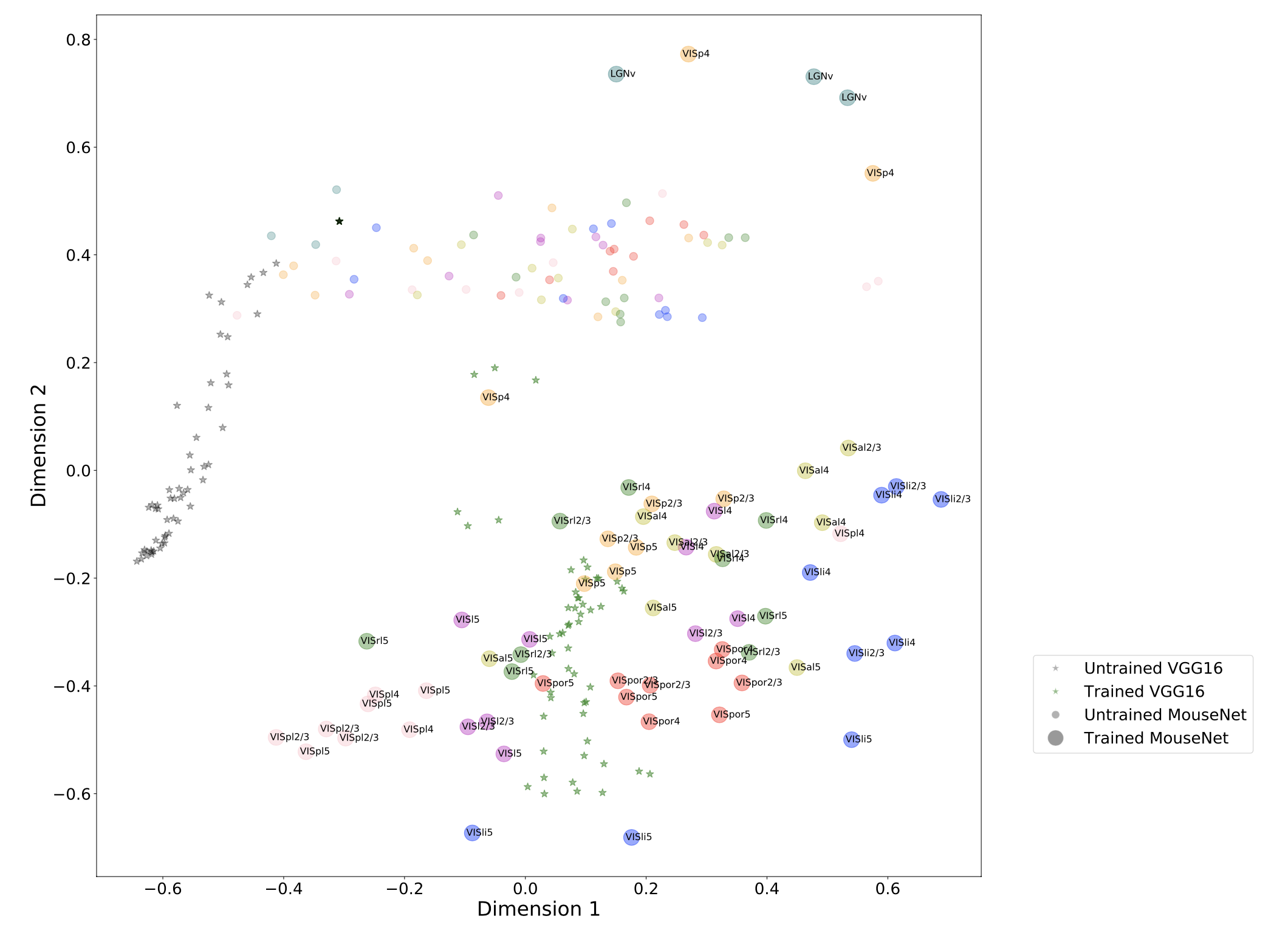

Supplement: S4 Fig — Each dot represents a layer from a certain model instance. The position of the dots are the two-dimensional projection from the multidimensional scaling algorithm, with the distance measure defined as one minus the SSM value. The layers from three instances of trained MouseNet are color coded by their area names, and annotated with their region names. This result shows that different pathways in the MouseNet have learned distinct representations after training. (TIF) [file pcbi.1010427.s007.tif]

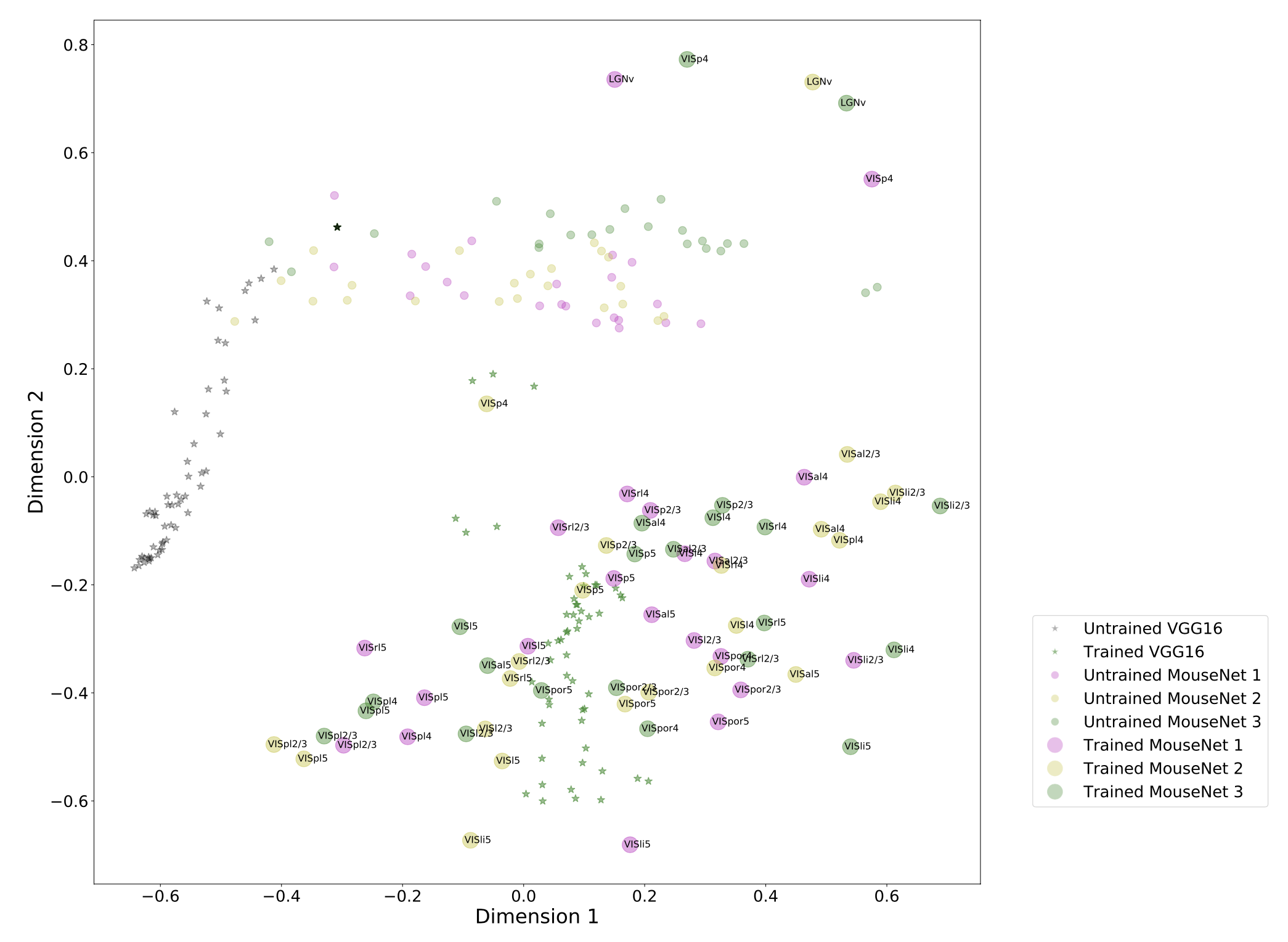

Supplement: S5 Fig — Each dot represents a layer from a certain model instance. The position of the dots are the two-dimensional projection from the multidimensional scaling algorithm, with the distance measure defined as one minus the SSM value. The layers from three instances of trained MouseNet are color coded by their corresponding model instance. This result shows that training diversified the representations of all the three instances of MouseNet starting from different initialization states. (TIF) [file pcbi.1010427.s008.tif]
